# Supplementary material for: UV-degraded polyethylene exhibits variable charge and enhanced cation adsorption
Source: PLoS One. 2025 Nov 21;20(11):e0337180. doi: 10.1371/journal.pone.0337180 (PMC12637955; doi:10.1371/journal.pone.0337180)
Supplement: S1 Table — (PDF) [file pone.0337180.s001.pdf]

S1 Table. Particle size distribution table of PE and PET after 0, 400, and 2000 hours of degradation.

plastic	mean ± s.d. [μm]	max peak ± s.d. [μm]	width ± s.d. [μm]	range [μm]	
				low	high
PE 0 h	375 ± 117	349 ± 15	189 ± 48	161	689
PE 400 h	370 ± 105	357 ± 17	223 ± 61	160	693
PE 2000 h	7.8 ± 7.5	4.5 ± 0.5	5.9 ± 2.3	< 1	66
PET 0 h	653 ± 219	519 ± 106	740 ± 454	333	1239
PET 400 h	531 ± 196	449 ± 20	298 ± 55	256	1286
PET 2000 h	484 ± 274	405 ± 41	640 ± 177	24	1165
