# Supplementary material for: UV-degraded polyethylene exhibits variable charge and enhanced cation adsorption
Source: PLoS One. 2025 Nov 21;20(11):e0337180. doi: 10.1371/journal.pone.0337180 (PMC12637955; doi:10.1371/journal.pone.0337180)
Supplement: S1 Fig — For non-polar/ non-dissociating surfaces, the isoelectric point (IEP) is determined around pH 4. (PDF) [file pone.0337180.s002.pdf]

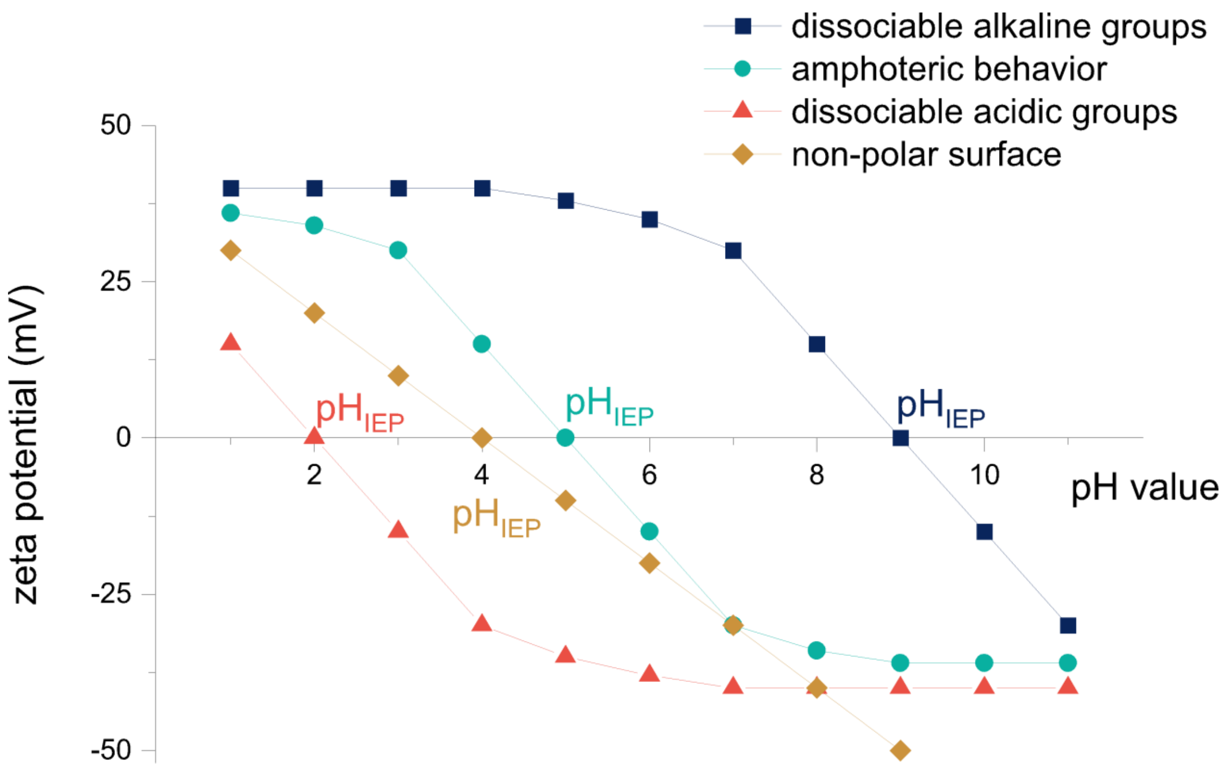

S1 Fig. Zeta potential (ζ) of particles with different functional groups in the presence of KCl under different pH conditions. For non-polar / non-dissociating surfaces, the isoelectric point (IEP) is determined around pH 4.
