# Supplementary material for: UV-degraded polyethylene exhibits variable charge and enhanced cation adsorption
Source: PLoS One. 2025 Nov 21;20(11):e0337180. doi: 10.1371/journal.pone.0337180 (PMC12637955; doi:10.1371/journal.pone.0337180)
Supplement: S2 Fig — Significant differences from Tukey HSD tests are displayed as different characters. (PDF) [file pone.0337180.s003.pdf]

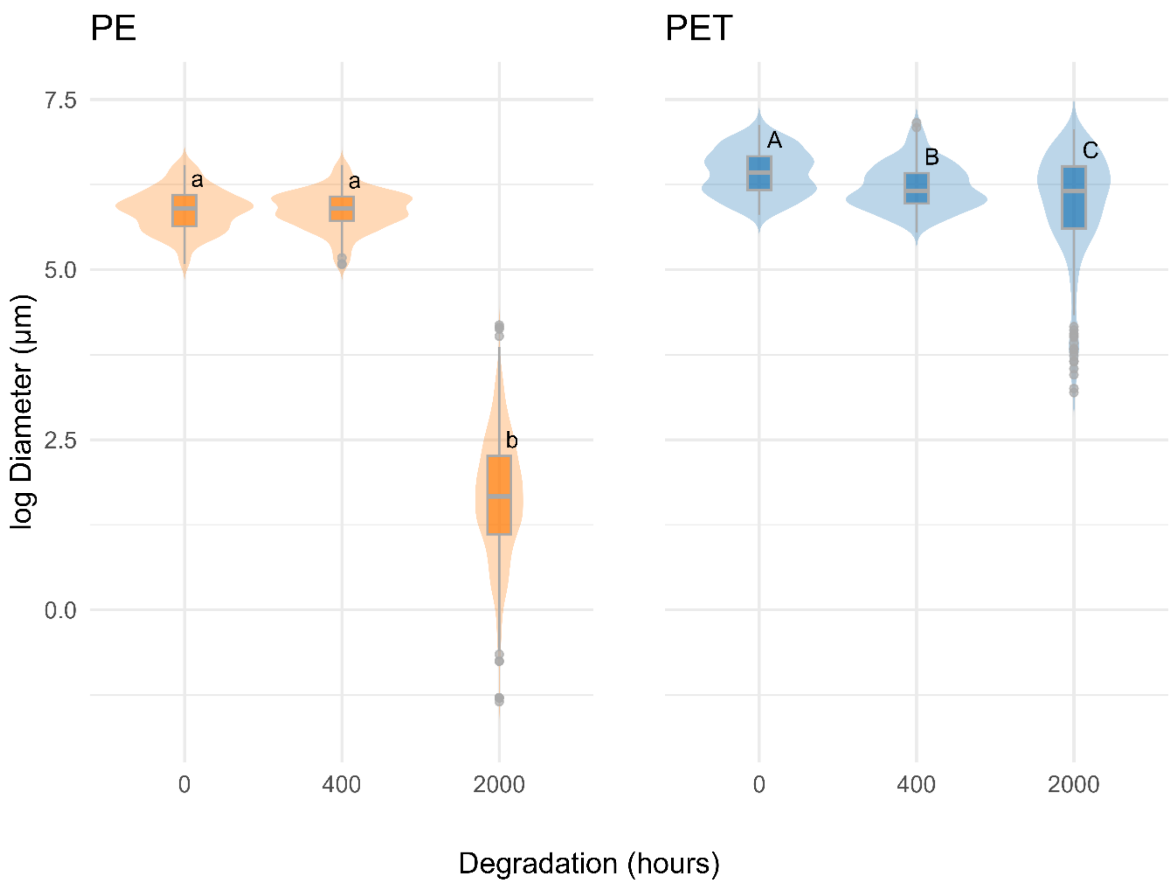

S2 Fig. Particle size distribution of PE and PET plastics after degradation (log-transformed). Significant differences from Tukey HSD tests are displayed as different characters.
