# Supplementary material for: UV-degraded polyethylene exhibits variable charge and enhanced cation adsorption
Source: PLoS One. 2025 Nov 21;20(11):e0337180. doi: 10.1371/journal.pone.0337180 (PMC12637955; doi:10.1371/journal.pone.0337180)
Supplement: S3 Fig — (PDF) [file pone.0337180.s004.pdf]

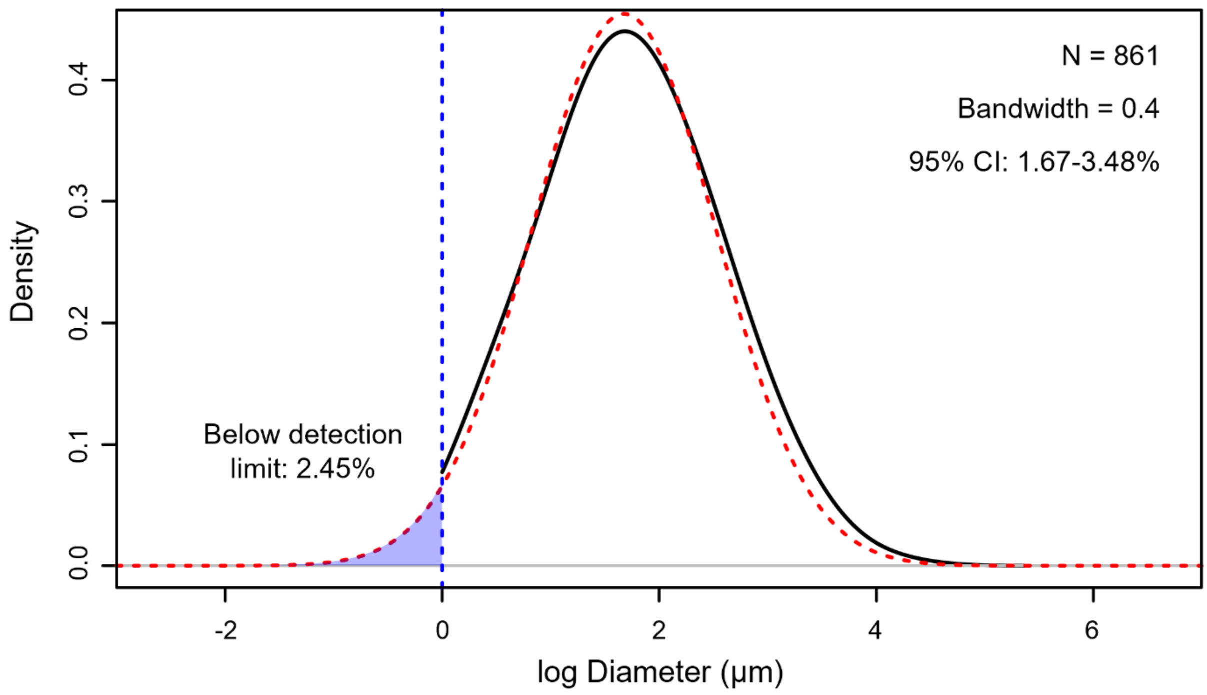

S3 Fig. PE 2000 hours degraded observed particle size distribution (log-transformed; black line), with estimated fit of truncated normal distribution (red dashed line) which estimated 2.5% with a confidence interval of 1.7-3.5% of the distribution as undetected particles <1 μm (blue dashed line).
