# Supplementary material for: UV-degraded polyethylene exhibits variable charge and enhanced cation adsorption
Source: PLoS One. 2025 Nov 21;20(11):e0337180. doi: 10.1371/journal.pone.0337180 (PMC12637955; doi:10.1371/journal.pone.0337180)
Supplement: S4 Fig — (PDF) [file pone.0337180.s005.pdf]

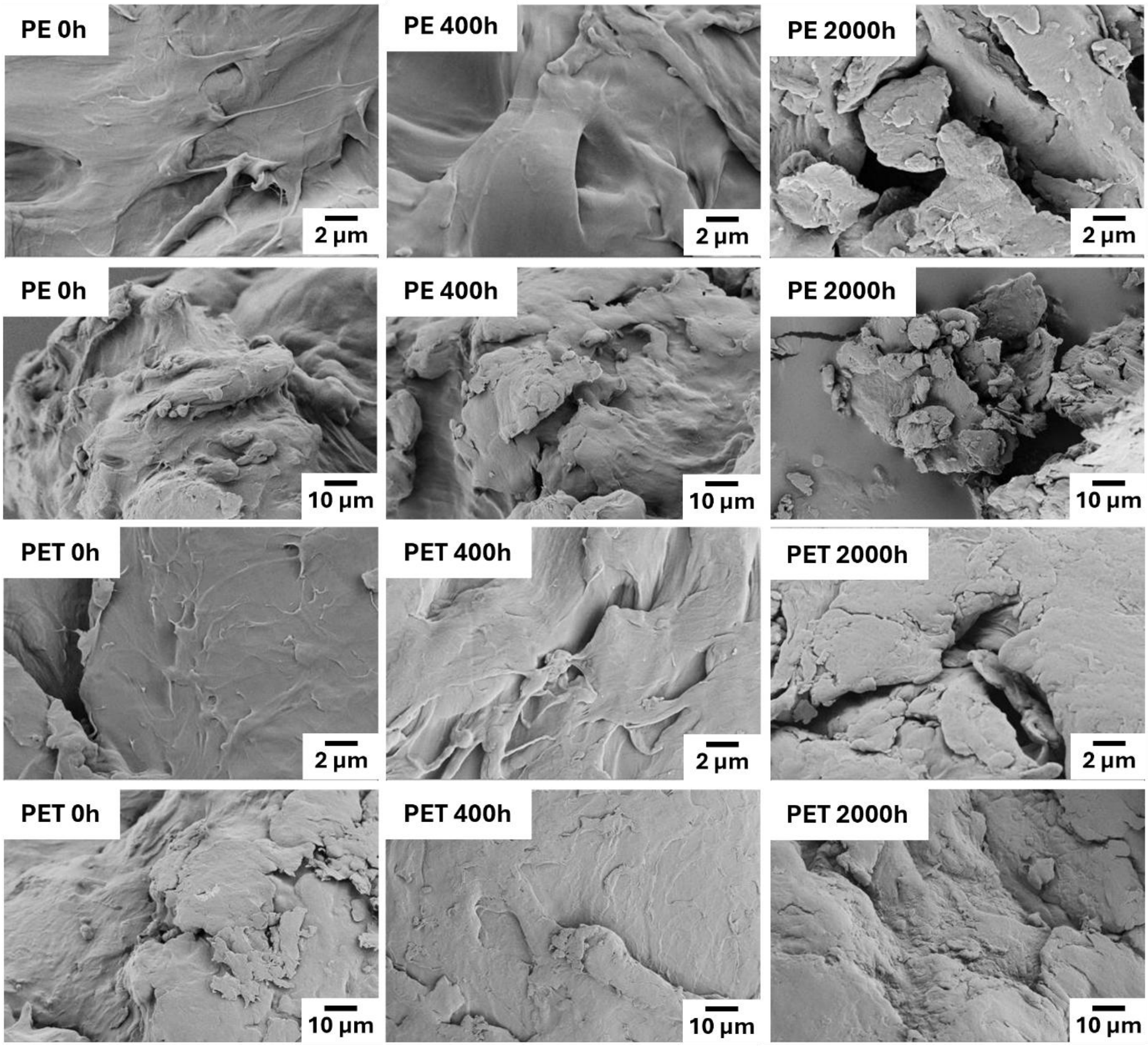

S4 Fig. SEM images of PE (top two rows) and PET (bottom two rows), pristine (0 h) and degraded at 400 h and 2000 h (left to right), at 5000x and 1000x magnification.
