# Supplementary material for: UV-degraded polyethylene exhibits variable charge and enhanced cation adsorption
Source: PLoS One. 2025 Nov 21;20(11):e0337180. doi: 10.1371/journal.pone.0337180 (PMC12637955; doi:10.1371/journal.pone.0337180)
Supplement: S5 Fig — PET particles show no significant changes in wettability over degradation time. (PDF) [file pone.0337180.s006.pdf]

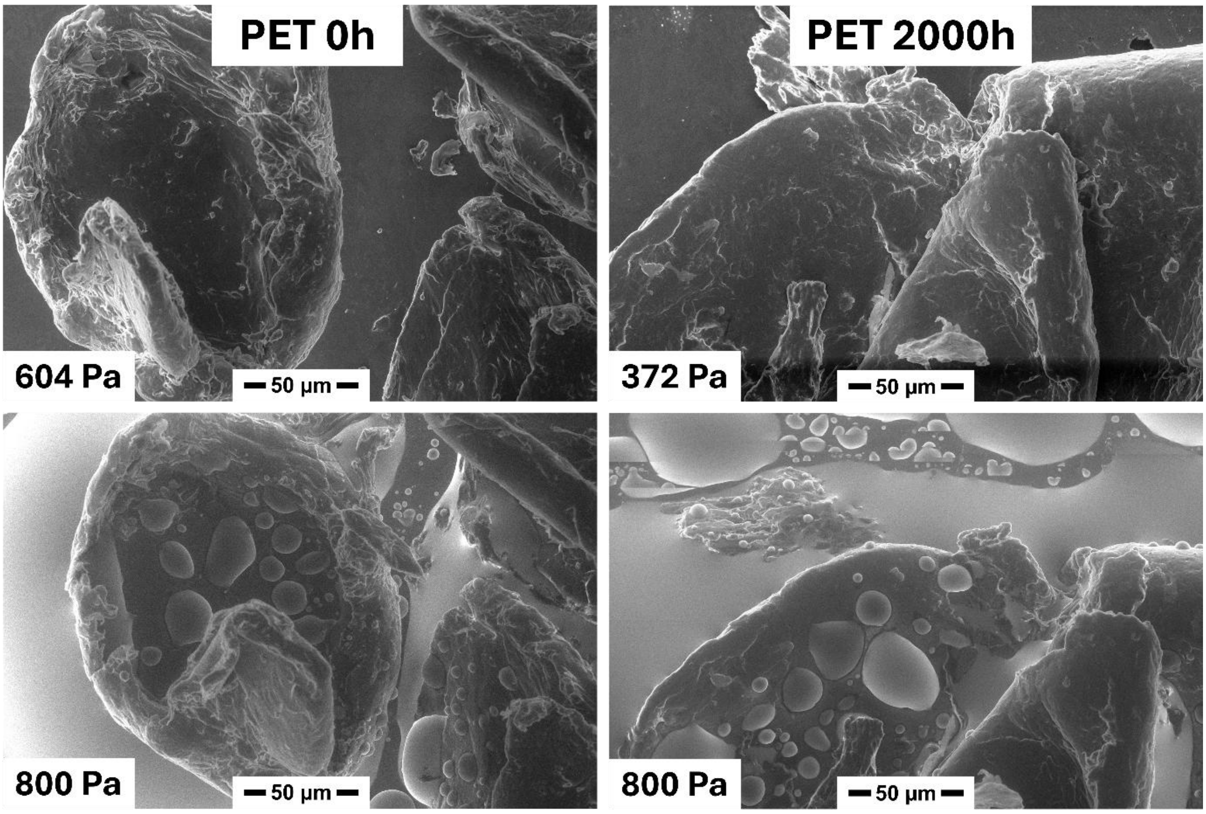

S5 Fig. ESEM images of PET pristine (left) and degraded at 2000 hours (right), at 300x magnification. PET particles show no significant changes in wettability over degradation time.
