# Supplementary figures and images for: UV-degraded polyethylene exhibits variable charge and enhanced cation adsorption
Source: PLoS One. 2025 Nov 21;20(11):e0337180. doi: 10.1371/journal.pone.0337180 (PMC12637955; doi:10.1371/journal.pone.0337180)

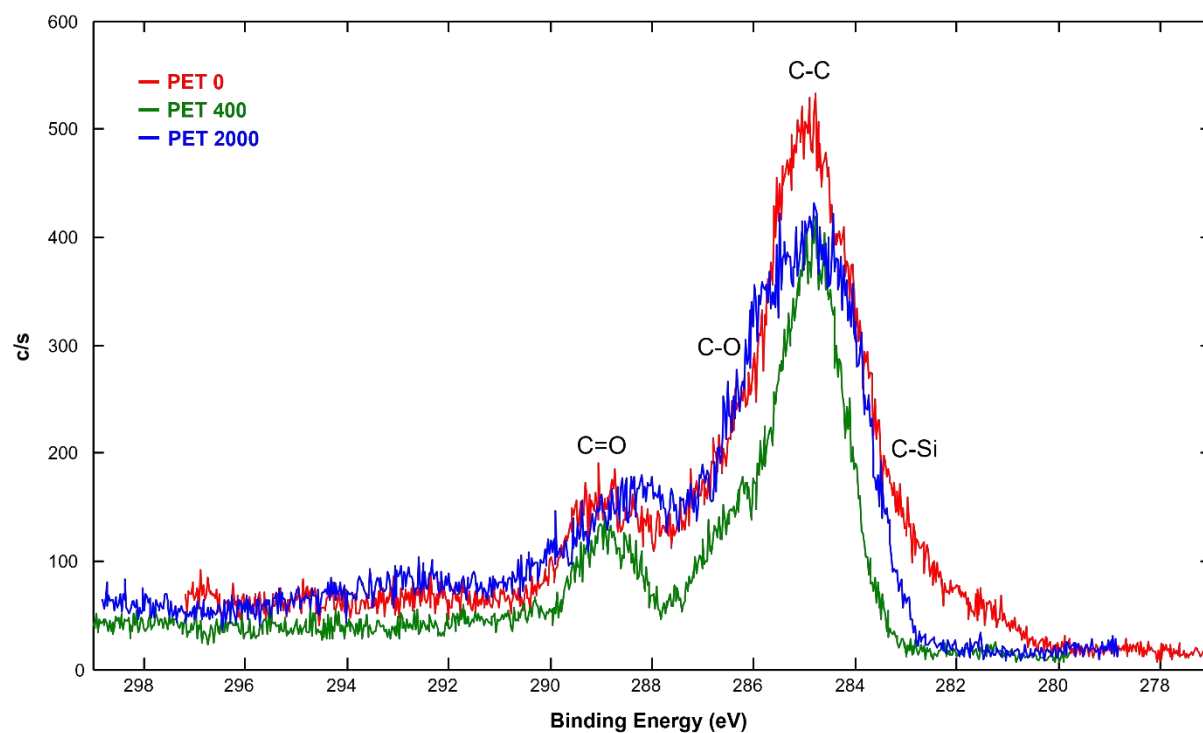

**S6 Fig.** XPS overlay of C1s spectra of PET degraded at 0 (red), 400 (green), and 2000 (blue) hours.

Supplement: S6 Fig — (PDF) [file pone.0337180.s007.pdf]
