# Supplementary material for: UV-degraded polyethylene exhibits variable charge and enhanced cation adsorption
Source: PLoS One. 2025 Nov 21;20(11):e0337180. doi: 10.1371/journal.pone.0337180 (PMC12637955; doi:10.1371/journal.pone.0337180)
Supplement: S7 Fig — Functional group formation progresses from alcohols to carbonyl and carboxylic acids, with decreased particle size and hydrophobicity, and increased surface area, roughness, charge, and ion binding capacity. (PDF) [file pone.0337180.s008.pdf]

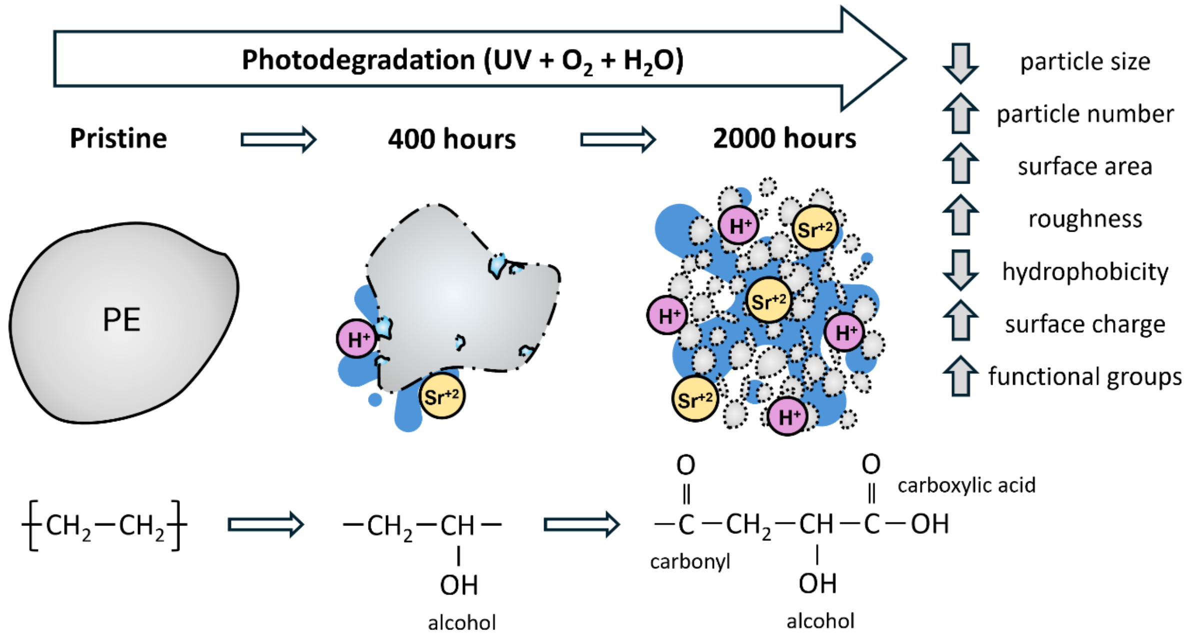

S7 Fig. Summary of PE degradation after 2000 hours of accelerated UV exposure (approx. 14 months natural sunlight) with proposed functional oxidation of polymer groups shown below. Functional group formation progresses from alcohols to carbonyl and carboxylic acids, with decreased particle size and hydrophobicity, and increased surface area, roughness, charge, and ion binding capacity.
